# Global dimensions of chronic kidney disease of unknown etiology (CKDu): a modern era environmental and/or occupational nephropathy?

**DOI:** 10.1186/s12882-015-0105-6

**Published:** 2015-08-19

**Authors:** Virginia M. Weaver, Jeffrey J. Fadrowski, Bernard G. Jaar

**Affiliations:** 1 Department of Environmental Health Sciences, Johns Hopkins University Bloomberg School of Public Health, Baltimore, MD USA; 2 Department of Medicine, Johns Hopkins Medical Institutions, Baltimore, MD USA; 3 Welch Center for Prevention, Epidemiology and Clinical Research, Johns Hopkins Medical Institutions, Baltimore, MD USA; 4 Department of Pediatrics, Johns Hopkins School of Medicine, Baltimore, MD USA; 5 Department of Epidemiology, Johns Hopkins University Bloomberg School of Public Health, Baltimore, MD USA

Diabetes and hypertension are the predominant risk factors for chronic kidney disease (CKD) globally. Infectious diseases resulting in glomerulonephritis are also important in low-income countries [1, 2]. However, in the past two decades, a severe form of CKD has been reported in individuals without these risk factors. CKD of unknown etiology (CKDu) affects adults in their third to fifth decade and is often fatal due to disease progression and lack of dialysis or transplant options in the involved geographic areas. CKDu has been reported in Sri Lanka, several Central American countries, the state of Andhra Prakesh in India and the El-Minia Governorate in Egypt.

The results of a joint Sri Lankan and World Health Organization (WHO) funded study focused on identification of risk factors for CKDu in Sri Lanka was recently published in this journal [3]. Subsequent correspondence, highlighting the range of risk factors under consideration, illustrates the complexity of this endeavor [4, 5]. Despite the comprehensive exposure assessment of Jayatilake and colleagues, and prior work in Sri Lanka and Central America, the name CKDu remains appropriate because the etiology is still unknown. Consideration of the similarities and differences in CKDu in the four regions in which it has been reported to date may be useful in the effort to determine causality and develop prevention strategies. Therefore, the table included in this manuscript compares and contrasts the currently available information on CKDu. Importantly, there is no global definition for this disease. Furthermore, levels of proteinuria and albuminuria tend to be low in Central America [6], however the case definition in Sri Lanka was based on persistent albuminuria defined as an albumin–creatinine ratio ≥30 mg/g in an initial urine sample and at a repeat visit [3]. Therefore, the data below utilize the criteria for CKDu of the authors in each publication.

Given the limited information about CKDu in the affected areas, particularly in Andhra Pradesh and Egypt, it is not clear that the etiology of the kidney disease is the same in all locations. Different risk factors have been emphasized; altitude and occupational risks factors have received more attention in Central America whereas extensive chemical monitoring was recently reported in Sri Lanka [3]. Moreover, case ascertainment in Sri Lanka has relied much more on proteinuria whereas, in Central America, both urine dipstick and serum creatinine have been used in case identification. Data on magnitude remains extremely limited.

However, as shown in the table, a number of similarities are present. The disease is characterized by substantial morbidity and mortality, resulting in death in young and middle aged adult patients, and absence of known causes of CKD such as diabetes and hypertension (or at least severe hypertension as the blood pressure inclusion criteria in the Sri Lankan study was less than 160/100 [3]). Men seem to be more at risk, at least for the most severe disease, and very poor rural areas are most affected with agricultural work being the dominant occupation. Poverty with lack of access to health care makes determining clinical characteristics of CKDu difficult. Even determining when the outbreaks actually started is challenging; CKDu may have been present for a significant period of time but not identified due to absence of diagnostic testing. Furthermore, many of these areas have recently seen a transition from earlier deaths from infectious diseases to deaths from non-communicable diseases in the setting of longer life spans which provides time for CKD to develop and progress.

Few studies have reported on urinary findings in CKDu patients. However, in described clinical presentations, typical patients present with a bland urine sediment and minimal proteinuria (Table 1). Recent studies have measured levels of kidney early biological effect markers in urine [7, 8]. The results are consistent with proximal tubular damage and may be useful in future work to identify CKDu at earlier stages and to determine etiology. Information on renal pathology has also been very limited to date. The two studies reporting biopsy results from patients with CKDu in El Salvador both found interstitial fibrosis with varying degrees of tubular atrophy, essentially proportional to stage of CKD. Both series also reported non-specific glomerular damage. Whether this is secondary to a primary tubulointerstitial process or represents a primary glomerular disease or could be due to recurrent ischemia of the glomerular capillaries, such as from dehydration or concomitant use of nonsteroidal anti-inflammatory drugs (NSAIDs), remains unknown. Wijkström et al. concluded that the biopsy results from Central America did not resemble any other common kidney disease [9]. Importantly, these findings are similar to those described in CKDu from Sri Lanka [10–12]. It would be very useful to have additional information on pathology, including at earlier stages of the disease process.

**Table 1:** Comparisons of Key Characteristics Among Areas with Reported CKDu

Risk Factor/Characteristic	Sri Lanka	Central America	India	Egypt
Reported Areas	North Central Province [3]	Most reports from El Salvador and Nicaragua but appears to extend across Pacific coast areas of Central America [37]	In state of Andhra Pradesh: coastal in Uddanam area and 30–40 km inland in Chimakurthy mandal [52]	Reported in El-Minia Governorate [53]
	Present, although to a lesser extent, in Uva and North Western Provinces [36]		In India overall, highest in south which included Andhra Pradesh [44]	
Age	Wide age range; increased prevalence of eGFR ≤ 60 ml/min per 1.73 m^2^ in fourth and fifth decades [12]	Third to fifth decade [37]	In India overall, younger than patients with diabetic nephropathy [44]	Mean age of 46 (*n* = 800 patients on renal replacement therapy) [53]
Sex	Female > male overall but male > female for CKD stage III –IV [3]	Male > female [37]	Male > female in Uddanam area, [6] and in India overall [44]	Male > female [53]
Geographical Characteristics	Rural [12]	Rural, especially the lowlands along the Pacific coast [37]	Rural - coastal and inland [52]	Rural [53]
	Dry weather except for two monsoon periods [36]	Coastal communities at lower elevations (<500 m) [45]		
Occupations	Chena (vegetable and other crops) farmers; rice farming had a lower risk compared to chena farming [3]	Risk in coastal agricultural workers but not in agricultural workers employed at elevations > 500 m; sugarcane workers studied in both locations [45]	In Uddanam area, agricultural cultivation of coconuts, rice, jackfruit and cashews [6]	Farming [54]
		Compared to coastal agricultural workers, risk lower in service sector and agricultural workers at higher elevations [55]		
		Intense heat noted in working conditions in Central America [49]		
Socio-economic Status	Low	Low	In India overall, lower than those with diabetic nephropathy [44]	Not reported
Pathology	In biopsies from 211 CKDu patients, the main pathological features were interstitial fibrosis, interstitial inflammation and tubular atrophy of varying degrees [10]. Authors concluded that interstitial fibrosis was the earliest detectable pathological change.	A study of 57 CKDu patients observed chronic tubulointerstitial nephropathy [56]. The authors considered the glomerular and vascular damage also observed to be secondary to the tubulointerstitial damage.	Chronic tubulointerstitial nephritis (no details as reported in abstract from conference proceedings) [6]	Not reported, biopsies rarely performed [53]
	Interstitial fibrosis and tubular atrophy, sometimes with nonspecific interstitial mononuclear cell infiltration, predominated; glomerular sclerosis, glomerular collapse, and features of vascular pathology such as fibrous intimal thickening and arteriolar hyalinosis also common (*n* = 57) [11]	A study of 8 CKDu patients reported extensive glomerulosclerosis (29 %-78 %) and signs of chronic glomerular ischemia in combination with tubular atrophy and interstitial fibrosis but only mild vascular lesions [9]. The authors concluded that both glomerular and tubulointerstitial compartments were damaged by CKDu.		
	Biopsies in 26 patients (19 in CKD stages 1–3) reported as consistent with tubulointerstitial disease; immunofluorescence tests for immune-mediated kidney injury were negative [12]			
Presentation	Slow progression; minimal proteinuria (mean 24 h urine protein = 612.8 mg in 109 participants) without active sediment; bilateral small echogenic kidneys [12]	Minor or no proteinuria or albuminuria [6, 55]	In India overall, advanced CKD, few initial symptoms, absent or mild hypertension and little or no proteinuria [44]	Not reported
	Urinary excretion of alpha-1-microglobulin elevated in CKDu patients, even in the earliest CKD stage, compared with first-generation related controls residing in the same community and Japanese controls, suggesting early renal tubular damage in CKDu [57]	Small echogenic kidneys on ultrasound [37]	In Uddanam area, proteinuria prevalence of 20 % in males and 12 % in females [6]	
		Urinary symptoms, when present, are positive for pyuria and leukocyte esterase but urine culture negative [37]		
Magnitude	Age-standardized prevalence (95 % CI) of albumin–creatinine ratio ≥30 mg/g on two separate tests [3]:	Mortality from chronic renal failure (2007) [58]	CKDu is second most common cause of CKD in India (16.0 %) after diabetic nephropathy (31.3 %) [44]	Unknown etiology, at 27 %, was leading cause of end-stage renal disease (ESRD) followed by hypertension at 20 % and glomerulonephritis at 11 % [53]
		El Salvador		
	15.1 % in Anuradhapura	Men: 85.5/100,000		
	20.6 % in Polonnaruwa	Women: 34.1/100,000		
		Nicaragua		
	22.9 % in Badulla	Men: 66.2/100,000		
	16.9 % (15.5 %–18.3 %) in women	Women: 22.3/100,000		
		USA		
	12.9 % (11.5 %–14.4 %) in men	Men: 9.5/100,000		
	Stage 3 and 4, respectively:	Women: 7.0/100/000		
		Cuba		
	23.2 % and 22 % in men	Men: 3.0/100,000		
	7.4 % and 7.3 % in women	Women: 2.5/100,000		

## Etiologic factors involved in past CKD outbreaks or poisoning due to nephrotoxicants

A review of investigations to determine etiologic factors involved in past end-stage renal disease (ESRD) outbreaks may also prove informative. Consider, for example, that the cause of Balkan endemic nephropathy remained a mystery for 50 years until the occurrence of nephropathy in individuals ingesting herbal preparations for weight loss led to the identification of aristolochic acid as the cause of both diseases [13]. Chronic interstitial nephritis is the most common pathologic diagnosis in CKD attributed to occupational and/or environmental exposures. It has been reported following excessive exposure to lead, cadmium, and aristolochic acid. Pathology consistent with this diagnosis is also present in biopsies in El Salvador and Sri Lanka although it is not the only finding on biopsy.

### Lead

**Fig. 1:**
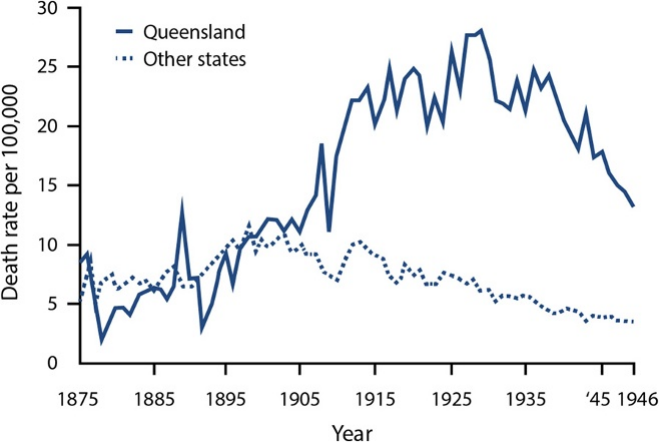
Annual age specific chronic nephritis mortality rates in persons under forty years of age in Queensland and the other States of Australia. Adapted from Figure 4 from Henderson DA: Chronic nephritis in Queensland. Australas Ann Med 1955, 4(3):163–177. Used with permission from Wiley

The historical outbreak that is most similar to CKDu in terms of mortality occurred in Queensland, Australia residents who survived lead poisoning as children but went on to die of ESRD as adults [14]. Lead paint was used in Queensland from 1890 until it was linked to lead poisoning in children and phased out, starting with a ban in 1922 [14]. Children were exposed through play on painted verandas and railings of raised houses; a type of housing unique to Queensland [14]. As shown in the Fig. 1 below, an epidemiological investigation of mortality from chronic nephritis revealed a dramatic spike in deaths starting in 1905 and peaking in the early 1930s [15].

In autopsy data, the content of lead in skull bone was higher in those who were born in Queensland and died in their third to fifth decade from ESRD of unknown cause with granular contracted kidneys compared to those who died of ESRD from known causes, such as chronic glomerulonephritis, and those who died from non-renal causes [16, 17]. Furthermore, levels of chelatable lead in urine were also higher in patients with CKD consistent with lead nephropathy [18].

However, in studies of adults who were lead poisoned as children in other geographic areas, increased mortality from ESRD and/or disease severe enough to require dialysis was uncommon. Only persistent partial Fanconi syndrome [19] and a few cases of kidney disease consistent with lead nephropathy have been reported [20, 21]. Differences in the extent of exposure may be involved since the Queensland children were not treated with chelation therapy. Researchers in the Queensland outbreak also implicated the additional impact of concentrated urine in children in this warm climate [16].

Higher ESRD mortality or incidence rates have also been reported in lead workers, most of whom were likely very highly exposed [22, 23]. However, the number of workers with a nonmalignant renal cause of death was still small [24] compared to residents in Queensland and in areas with CKDu. Furthermore, blood lead is easy to measure and elevated levels have not been detected in Sri Lanka [3] or in Central America [25]. Thus, lead exposure is not a likely explanation for CKDu.

### Cadmium

Excessive cadmium exposure has occurred in several areas in Japan but is best known as the cause of Itai-itai (“ouch-ouch”) disease in the Jinzu River basin of Toyama prefecture. Ingestion of rice irrigated with industrially polluted water resulted in high level exposure to cadmium. One study reported mean urine cadmium levels of 25.6 and 36.7 μg/g creatinine in 34 males and 38 females, respectively [26]. High levels were also observed in other studies [27]. To put these levels in context, the geometric mean urine cadmium in the U.S. National Health and Nutrition Survey was 0.25 μg/g creatinine in the 2007–2008 survey [28]. The most obvious adverse health effects from the Japanese exposure, reflected in the disease name, were osteomalacia and osteoporosis with secondary fractures and severe pain. Older women were most affected; this is attributed to their poor nutritional status, especially following World War II, since iron deficiency increases cadmium absorption. Proximal tubule damage was also a common feature of this disease and decreased creatinine clearance was reported in affected residents in the Jinzu river basin and other cadmium polluted areas in Japan [26, 29, 30]. However, despite high levels of exposure based on cadmium measurement in urine and rice, kidney disease severe enough to result in early death was not commonly reported. Four clinically identified deaths from uremia in Itai-itai patients have been described in the literature [30]. Studies among inhabitants living in the cadmium polluted area of the Kakehashi river basin observed increased mortality from nephritis and nephrosis, also based on small numbers [31].

In a more recent environmental exposure in the Mae Sot district of Thailand, evidence of proximal tubular damage is present but no outbreak of ESRD requiring dialysis or resulting in death has been reported in the affected population with mean urine cadmium levels of 5 μg/g creatinine [32]. A general population study in Belgium reported an association between higher blood lead and lower creatinine clearance; no associations with this outcome were observed with urine or blood cadmium [33]. A similar situation was observed in occupational exposure. Even in exposure conditions considered extremely high by current standards, tubular damage and CKD occurred but were rarely severe enough to progress to ESRD [34, 35].

The recently reported values of 1.04 and 0.65 μg/g creatinine in participants with CKDu and controls in the Sri Lanka endemic area, respectively, were much lower than in any of the studies described above [3]. Thus, it is unlikely that cadmium is the sole cause of CKDu in Sri Lanka.

### Aristolochic acid

Aristolochic acid, a naturally occurring nephrotoxic compound found in plants of the genus *Aristolochiaceae*, has been implicated as the cause of interstitial nephritis from ingestion of Chinese herbal medications used for weight loss that were contaminated with this compound [13]. Aristolochic acid has also been implicated in Balkan endemic nephropathy via plants growing around wheat fields in affected areas [13]. CKD from aristolochic acid ingestion does progress to ESRD, within 2–3 years in the case of herb ingestion. However, both aristolochic acid nephropathy and Balkan endemic nephropathy are associated with increased risk for urothelial carcinoma which has not been reported in CKDu to date. Further, data to date, although limited, do not indicate greater exposure to this chemical in the affected areas compared to non-endemic surrounding areas [36, 37].

### Arsenic

Elevated standardized mortality rates for kidney disease have been reported in ecologic studies of communities with moderate to high levels of arsenic in their water sources [38]. Reduction in mortality after cessation of exposure in a previously endemic area in Taiwan was also observed [39]. A recent longitudinal study reported an increased risk for incident CKD with baseline urine arsenic concentrations [40]. However, similar to the situation with cadmium, reported arsenic levels in water [3] were much lower in Sri Lanka than in the communities where increased risk was reported in the ecologic studies. Furthermore, mortality in CKDu is so high that it does not require a mortality study to detect it.

### Other environmental causes of kidney disease

Other exposure-related outbreaks have involved acute kidney injury. Ingestion of formula adulterated with melamine resulted in an outbreak in Chinese infants due to urinary tract obstruction from kidney stones. The long term implications of this exposure remain a research focus [41]. In a much smaller outbreak, diethylene glycol contaminated acetaminophen syrup caused acute kidney injury in Haitian children. The case fatality rate was extremely high (88 of 109 exposed children) [42]. However, the acute nature of these outbreaks is quite different than the pattern described to date in CKDu. Certain medications can also cause or contribute to CKD. Analgesic nephropathy is one of the best known examples of this; also requiring substantial research to make the connection.

### CKDu risk factors

Considering the CKDu characteristics reported globally and the environmental causes of CKD outbreaks identified to date, none of the previously established causes alone appears to explain CKDu. Thus, this disease entity is either multifactorial and/or due to a previously unrecognized cause of kidney damage. Two reviews have discussed the range of risk factors under consideration, in Central America [37] and globally [6]. As noted above, different risk factors may be involved in the various locations in which CKDu has been reported. Ethnic diversity may also be a factor. For example, South Asians have an increased risk for coronary artery disease that is not explained by traditional cardiac risk factors [43]. Given the similarities between cardiac and renal risk factors, this risk may be relevant for CKDu in India and Sri Lanka but not necessarily related to CKDu in Central America. An additional challenge for etiologic determination is that the majority of countries report at least some baseline rate of CKD that appears unrelated to traditional risk factors and thus is considered to be of unknown etiology [2]. As noted by Rajapurkar and colleagues, disease in which the cause could not be determined was the second most common CKD presentation in India and was observed throughout the country [44]. They note that delayed presentation in areas with limited access to healthcare makes establishing a primary diagnosis very challenging. Thus, there is likely some proportion of disease that is due to lack of diagnostic capability within the category of disease not due to any common risk factor that actually represents a novel type of CKDu.

Extreme physical exertion, heat stress, water quality and exposure to agrochemicals are among the potential causes currently being considered for CKDu. The combination of heat stress and physically demanding occupations has received attention in the Central American outbreak based on studies reporting lower rates of CKDu at higher altitudes in the same agricultural processes [45]. A recent pilot study in Brazilian sugarcane harvesters observed evidence of acute kidney injury over the course of a workday [46] The impact of repeated episodes of heat-induced dehydration has been examined in an animal model to specifically address this cause [47]. Pathology consistent with CKDu, including elevated serum creatinine, proximal tubular injury, and renal inflammation and fibrosis, was observed. Interestingly, this pathology was not observed in fructokinase deficient mice. Fructose containing drinks in combination with heat-induced dehydration was implicated and intervention trials based on the United States’ Occupational Safety and Health Administration (OSHA) Heat Illness Prevention Campaign were proposed [48]. In considering this potential etiologic factor, one must consider whether agricultural work has changed within the time frame of the CKDu epidemic. The industrialization of animal husbandry in the form of concentrated animal feeding operations has dramatically changed that field. Has the same magnitude of change occurred for agricultural workers? Work in high levels of ambient heat was common in a recent study of working conditions in Central America [49]. However, in a recent spatial distribution analysis, high temperature did not explain CKDu occurrence after adjustment for area under cultivation for specific crops [50]. Given the increased reliance on agrochemicals in modern agriculture, direct exposure and water contamination remain important considerations as well.

### Conclusions and recommendations

A form of CKD that appears unrelated to traditional risk factors, such as diabetes and hypertension, is responsible for widespread morbidity and mortality in specific geographic locations across three continents. The cause(s) remains unknown; however none of the occupational or environmental exposures that have been implicated in previous outbreaks of nephropathy have been established as sole risk factors in the current outbreaks. Thus, multifactorial or novel risks must be considered. Addressing similarities and differences in CKDu reported in different geographic areas globally may be of value in unraveling the cause(s) of this severe form of kidney disease.

Future needs include funding for clinical care of affected populations and for continued etiologic research. CKDu mortality is extremely high, reflecting health care limitations in the low income countries impacted by this disease. Therefore, funding for patient care is urgently needed. Funding for preventive medical care to identify kidney disease at earlier, more treatable stages is also necessary. In addition, funding is needed for basic public health measures that are known to be cost effective and also address potential CKDu risk factors. Examples include water sources that are free of biological and chemical contaminants; work practices that prevent dehydration in workers; and use of accepted safety procedures for agrochemicals. Implementation of these measures will benefit the population overall and may reduce the risk of CKDu since, based on experience with Balkan nephropathy, establishing the etiology may be a prolonged process.

In terms of research funding needs, in addition to continued etiologic studies, communication between research groups in the different geographic areas where CKDu has been reported would be useful to consider similarities and differences in the disease in each location as well as consolidate research approaches when possible. This would allow development of an internationally accepted definition of CKDu and kidney biopsy pathology criteria. Conference funding would be very valuable in this regard. Given the extremely high mortality and morbidity reported with this disease, efforts to identify the cause(s), prevent future cases and provide care for those affected must be a global priority.

**Table:** 

Resources for additional information include an issue devoted to CKDu in the MEDICC (Medical Education Cooperation with *Cuba*) Review: International Journal of Cuban Health and Medicine (http://www.medicc.org/mediccreview/index.php?issue=28). In addition, reflecting ongoing public concern for this serious disease, news organizations have reported on the outbreaks. The Center for Public Integrity and its International Consortium of Investigative Journalists has several articles and videos such as http://www.publicintegrity.org/health/mystery-fields. The Central American outbreak and challenges surrounding research related to it are discussed in a recent Science newsfocus [51].	

## Abbreviations

CKDu: Chronic kidney disease of unknown etiology
CKD: Chronic kidney disease
WHO: World Health Organization
NSAIDs: Nonsteroidal anti-inflammatory drugs
ESRD: End-stage renal disease
OSHA: Occupational Safety and Health Administration
